# Comparative analysis of human and mouse transcriptomes during skin wound healing

**DOI:** 10.3389/fcell.2024.1486493

**Published:** 2024-10-29

**Authors:** Maochun Wang, Jiao Zhang, Chongxu Qiao, Shunchao Yan, Guoping Wu

**Affiliations:** Department of Plastic Surgery, The Affiliated Friendship Plastic Surgery Hospital of Nanjing Medical University, Nanjing, Jiangsu, China

**Keywords:** comparative analysis, molecular network, skin wound healing, human transcriptomes, mouse transcriptomes

## Abstract

Skin wound healing is a complex process which involves multiple molecular events and the underlying mechanism is not fully understood. We presented a comparative transcriptomic analysis of skin wound healing in humans and mice to identify shared molecular mechanisms across species. We analyzed transcriptomes from three distinct stages of the healing process and constructed protein-protein interaction networks to elucidate commonalities in the healing process. A substantial number of differentially expressed genes (DEGs) were identified in human transcriptomes, particularly upregulated genes before and after wound injury, and enriched in processes related to extracellular matrix organization and leukocyte migration. Similarly, the mouse transcriptome revealed thousands of DEGs, with shared biological processes and enriched KEGG pathways, highlighting a conserved molecular signature in skin wound healing. A total of 21 common DEGs were found across human comparisons, and 591 in mouse comparisons, with four genes (KRT2, MARCKSL1, MMP1, and TNC) consistently differentially expressed in both species, suggesting critical roles in mammalian skin wound healing. The expression trends of these genes were consistent, indicating their potential as therapeutic targets. The molecular network analysis identified five subnetworks associated with collagen synthesis, immunity, cell-cell adhesion, and extracellular matrix, with hub genes such as COL4A1, TLR7, TJP3, MMP13, and HIF1A exhibited significant expression changes before and after wound injury in humans and mice. In conclusion, our study provided a detailed molecular network for understanding the healing process in humans and mice, revealing conserved mechanisms that could help the development of targeted therapies across species.

## Introduction

Skin is the first protective barrier for human and animals, but due to constantly being challenged by various external environment, it is very susceptible to trauma. Skin wound healing is a highly complex physiological process which involves intracellular and intercellular mechanisms to restore skin tissue homeostasis after injury (Wilkinson and Hardman, 2020; Sorg et al., 2016). It is influenced by various risk factors, including oxygenation, infection, age, gender, stress, diabetes, obesity, medicine, alcoholism, smoking, and nutrition (Guo and DiPietro, 2010a; Guo and DiPietro, 2010b). This process is composed of hemostasis, inflammation, proliferation, and remodeling, and it is shown that many cells are involved, including platelets, neutrophils, macrophages, lymphocytes, fibroblasts, and epidermal cells (Singh et al., 2017; Cañedo-Dorantes and Cañedo-Ayala, 2019). However, dysregulation of any of these stages can lead to chronic non-healing wounds or hypertrophic scarring, such as venous leg ulcers and diabetes foot ulcers, which can significantly impact the quality of patient’s life (Wilhelm et al., 2017; Medina et al., 2005). Over the past few decades, the growing population and increased demand for surgical procedures have amplified the burden of wound care. Therefore, scientific and medical research interest in this field has grown significantly.

Advances in transcriptomics have provided new insights into the molecular mechanisms underlying wound healing. Recent studies using high-throughput RNA sequencing have provided a comprehensive view of the dynamic gene expression changes that occur throughout the different phases of wound healing, from the inflammatory response to tissue remodeling (Rong and Liu, 2020; Chen et al., 2022). For instance, transforming growth factor-beta (TGF-β) and fibroblast growth factors (FGFs) family members have shown to regulate fibroblast activity and collagen deposition, which are essential for the formation of extracellular matrix (Farooq et al., 2021; Nigdelioglu et al., 2016; Honda et al., 2012; Kashpur et al., 2013). Additionally, the role of inflammatory cytokines such as IL-1, IL-6, and TNF-α has been clarified, demonstrating their complex interactions in coordinating the immune response and subsequent tissue regeneration (Grellner, 2002; Zhou et al., 2020). Furthermore, single-cell RNA sequencing has allowed for the identification of distinct cell subpopulations within the wound microenvironment at single-cell resolution, each with unique gene expression profiles to understand the cellular and molecular events that contribute to the overall wound healing process (Haensel et al., 2020; Guerrero-Juarez et al., 2019). These techniques have not only deepened our knowledge of the molecular basis of wound healing, but also uncovered novel biomarkers and potential therapeutic targets to improve wound healing outcomes, particularly in chronic or non-healing wounds.

Here we used transcriptomic data from three distinct stages of skin wound healing in humans and mice to identify shared differentially expressed genes (DEGs) and the results provided insights to the shared and distinct molecular mechanisms underlying skin wound healing across species. The identification of conserved genes and networks not only provided a deeper understanding of skin wound healing process but also offered potential targets for therapeutic intervention.

## Material and methods

### Quality control of human and mouse transcriptomes

Transcriptomes of human and mouse during skin wound healing were collected from Gene Expression Omnibus (GEO) database, which the access number were GSE50425 and GSE113081 respectively. Human transcriptome contained four intact skin samples, four 14th post-operation day samples and four 21st post-operation day samples (hPWD0 = 4, hPWD14 = 4, hPWD21 = 4), which were collected from biopsies of patients undergoing split-thickness skin graft harvesting. Mouse transcriptome composed of three normal skin samples, four 7 days post-wounding samples and four 14 days post-wounding samples (mPWD0 = 3, mPWD7 = 4, mPWD14 = 4), which were generated from 8-mm skin wounds of adult mice (Brant et al., 2019). Raw data of human transcriptome from Illumina HumanHT-12 V4.0 expression beadchip was read and normalized by lumi (v2.52.0) R package, and probes were converted to symbols with expression values calculated by average method, which obtained 18,685 unique genes. Raw counts of mouse transcriptome from Illumina NextSeq 500 were read and normalized with DESeq2 (v1.40.2) R package, and counts were transformed to TPM to perform quality control, in which 16,576 unique genes were used for further analysis. Principal Component Analysis (PCA) of human and mouse transcriptome was conducted using factoextra (v1.0.7) and FactoMineR (v2.9) R packages. Association of top 500 highly expressed genes in each sample of human and mouse transcriptome were clustered by pheatmap (v1.0.12) R package.

### Transcriptome analysis

For the human transcriptome, three pairwise comparisons were made (hPWD14 vs hPWD0, hPWD21 vs hPWD0, and hPWD21 vs hPWD14), with differentially expressed genes (DEGs) identified using a threshold of 1.5-fold expression difference and P-value less than 0.05. As the mouse transcriptome was derived from RNA-seq, three groups of mouse transcriptome were compared pairwise respectively (mPWD7 vs mPWD0, mPWD14 vs mPWD0, and mPWD14 vs mPWD7) and we used relatively strict screening criteria with 2-fold expression difference and P-value less than 0.05. The ggplot2 (v3.4.4) R package was used to create gene volcano plots, with the top 10 DEGs highlighted. Top 20 DEGs were clustered by pheatmap (v1.0.12) R package. All DEGs were further enriched by Gene Ontology (GO) and Kyoto Encyclopedia of Genes and Genomes (KEGG) with clusterProfiler (v4.8.3), GOplot (v1.0.2) and enrichplot (v1.20.3) R packages. The DEGs of different groups and the shared genes in the human and mouse transcriptomes were displayed with venn diagrams using venn (v1.11) and VennDiagram (v1.7.3) R packages. Shared DEGs of the human and mouse skin wound healing were imported in STRING database and analyzed with Cytoscape (v3.10.1) to construct Protein-protein interaction (PPI) network. We used MCODE plugin with default parameters to decompose the important sub networks.

### Statistical analysis

Relative expression values of shared genes and hub genes were presented as mean ± SD, with one-way analysis of variance (ANOVA) method conducted in GraphPad Prism 9 (v9.3.1) for statistical analysis. ns indicated that there was no statistical significance. * was considered that there was statistical significance (*p < 0.05, **p < 0.01, ***p < 0.001, ****p < 0.0001).

## Results

### Skin wound healing of human and mouse transcriptomes

**FIGURE 1:**
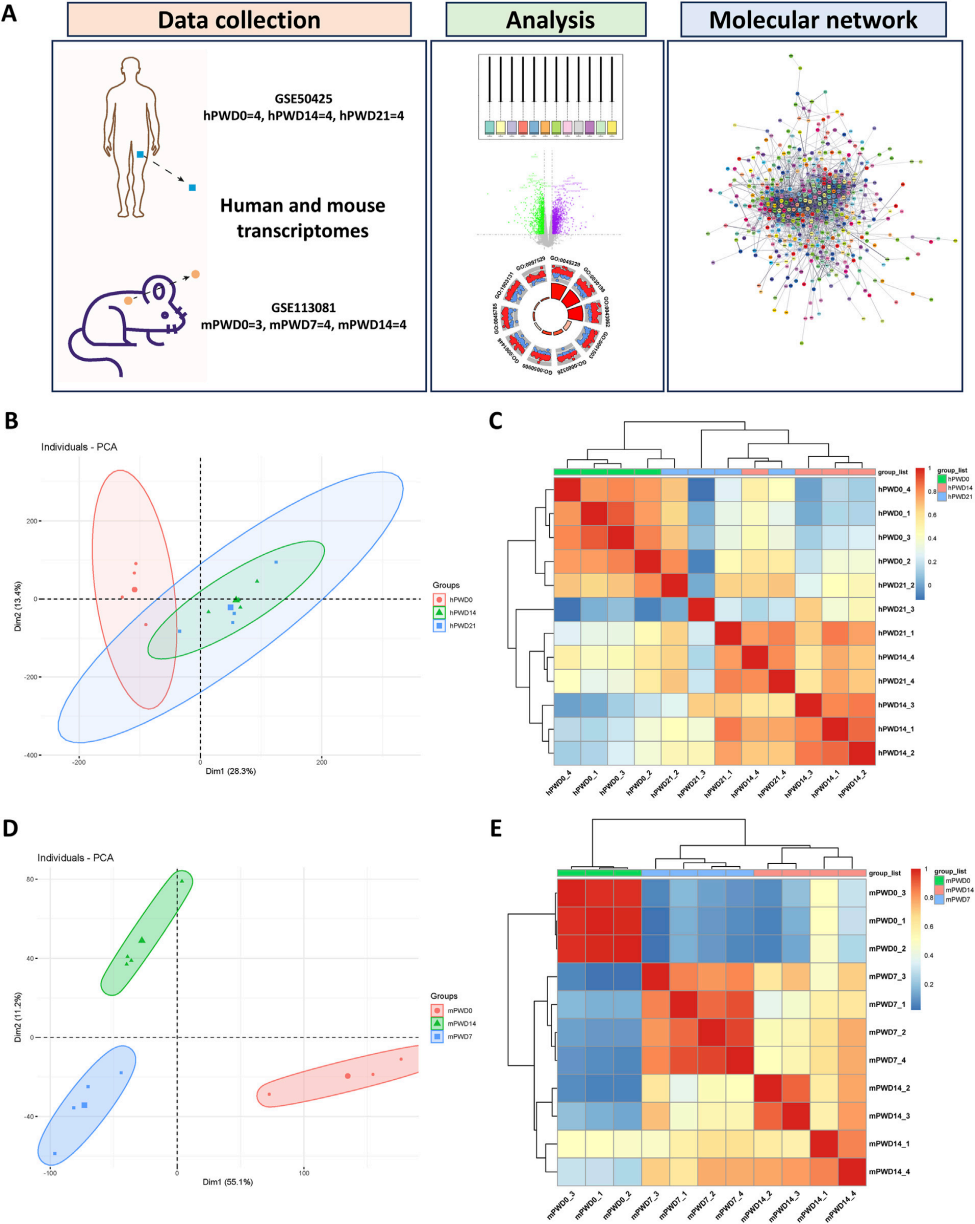
Cluster analysis of human and mouse transcriptomes during skin wound healing. **(A)** A schematic of this study. **(B)** Principal component analysis of human transcriptome. Circle represents hPWD0, triangle represents hPWD14 and square represents hPWD21. **(C)** Heatmap of correlation between hPWD0, hPWD14 and hPWD21. **(D)** Principal component analysis of mouse transcriptome. Circle represents mPWD0, triangle represents mPWD14 and square represents mPWD7. **(E)** Heatmap of correlation between mPWD0, mPWD7 and mPWD14. Association of top 500 highly expressed genes in each sample of human and mouse transcriptome were clustered by pheatmap (v1.0.12) R package.

Overall, we utilized transcriptomes from the three stages of skin wound healing in human and mice to screen for shared differentially expressed genes, and constructed protein-protein interaction network to reveal the common molecular mechanism involved in skin wound healing in both human and mice (Figure 1A).

We normalized human and mouse transcriptomes before analysis. Boxplot showed that the medians of data were relatively uniform in different datasets after normalization (Supplementary Figures S1A, C). PCA and cluster dendrograms revealed differences between the hPWD0 and the hPWD14, hPWD21 human transcriptomes, with notable similarities between hPWD14 and hPWD21 (Figure 1B; Supplementary Figure S1B). In contrast, mouse transcriptomes (mPWD0, mPWD7, mPWD14) clustered separately, indicating distinct group differences (Figure 1D; Supplementary Figure 1D). We further extracted 500 highly expressed genes from each dataset to test the correlation between samples, and found the correlation within hPWD0, hPWD14, and hPWD21 group of human transcriptomes, and the correlation within mPWD0, mPWD7, and mPWD14 of mice transcriptomes (Figures 1C, E). Meanwhile, we could also determine group similarity between hPWD14 and hPWD21, and although mouse mPWD7 and mPWD14 also showed partial similarity, they could be distinguished (Figures 1C, E). This could be due to individual variability in human samples or minimal transcriptomic changes in the later stages (hPWD14 and hPWD21) of human skin wound healing.

### Skin wound healing of human transcriptome

**FIGURE 2:**
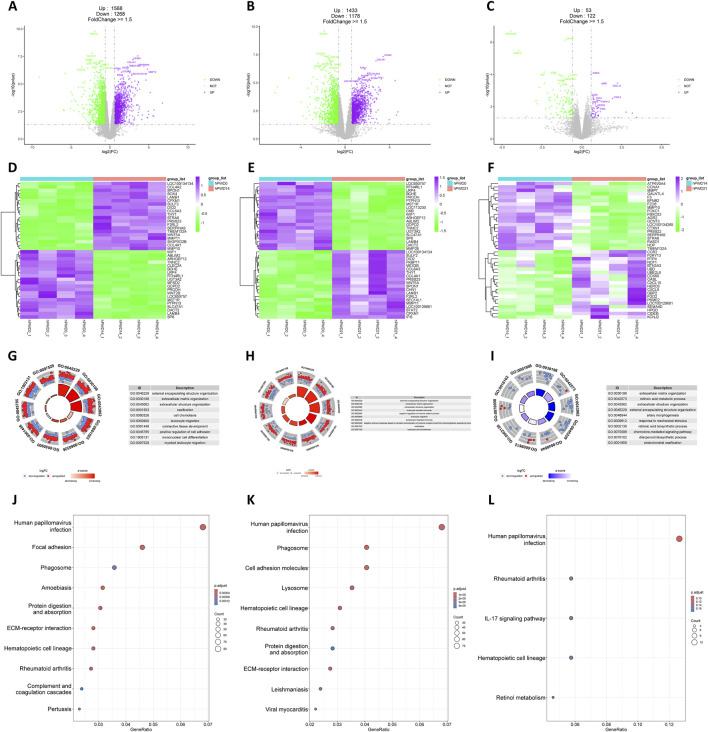
Transcriptomic analysis of human during skin wound healing. **(A–C)** Volcano plot of hPWD14 vs hPWD0 **(A)**, hPWD21 vs hPWD0 **(B)**, and hPWD21 vs hPWD14 **(C)** comparison. Purple indicates upregulated genes and green indicates downregulated genes. Top 20 differentially expressed genes are labeled in the map. **(D–E)** Heatmap of top 20 differentially expressed genes in hPWD14 vs hPWD0 **(D)**, hPWD21 vs hPWD0 **(E)**, and hPWD21 vs hPWD14 **(F)** comparison. **(G–I)** Biological process enrichment of differentially expressed genes in hPWD14 vs hPWD0 **(G)**, hPWD21 vs hPWD0 **(H)**, and hPWD21 vs hPWD14 **(I)** comparison. **(J–L)** Kyoto Encyclopedia of Genes and Genomes (KEGG) analysis of differentially expressed genes in hPWD14 vs hPWD0 **(J)**, hPWD21 vs hPWD0 **(K)**, and hPWD21 vs hPWD14 **(L)** comparison.

A substantial number of DEGs were identified in the hPWD14 vs hPWD0 and hPWD21 vs hPWD0 comparisons of the human transcriptome, with 2,836 and 2,611 DEGs, respectively, which there were more upregulated genes than downregulated genes (Figures 2A, B). In hPWD21 vs hPWD14, there were fewer DEGs (N = 175), while the majority of genes were downregulated (Figure 2C). Certain genes exhibited consistent expression changes in both hPWD14 and hPWD21 compared to hPWD0. LOC100134134, SPON1, LAMB1, CPXM1, SULF2, DIO2, COL6A3, THY1, PRSS23, F2RL2, WNT5A, MMP11, and COL4A1 showed increased expression, while WIF1, ABLIM2, ARHGEF12, TNNC2, BCHE, LRP4, RTN4L1, UGT3A2, GDPD2, PRODH, LOC650757, MST1R, PTPN13, SLC47A1, DACT2, LAMB4, and SP8 showed decreased expression (Figures 2D, E). These genes were not found in the comparison between hPWD21 and hPWD14 (Figure 2F). DEGs in hPWD14 and hPWD21 were enriched in wound healing related process, including external encapsulating structure organization, extracellular matrix organization, extracellular structure organization, ossification, and leukocyte migration (Figures 2G, H). While in hPWD21 vs hPWD14, DEGs were not only associated with extracellular matrix, but also associated with retinoic acid metabolic process (Figure 2I). KEGG analysis showed that DEGs were enriched in infection and cellular homeostasis in hPWD14 and hPWD21, like human papillomavirus infection, phagosome, protein digestion and absorption, and ECM-receptor interaction (Figures 2J, K). Most DEGs in the hPWD21 vs hPWD14 comparison were associated with infection, suggesting ongoing infection influence 21 days post-injury (Figure 2L).

### Skin wound healing of mouse transcriptome

**FIGURE 3:**
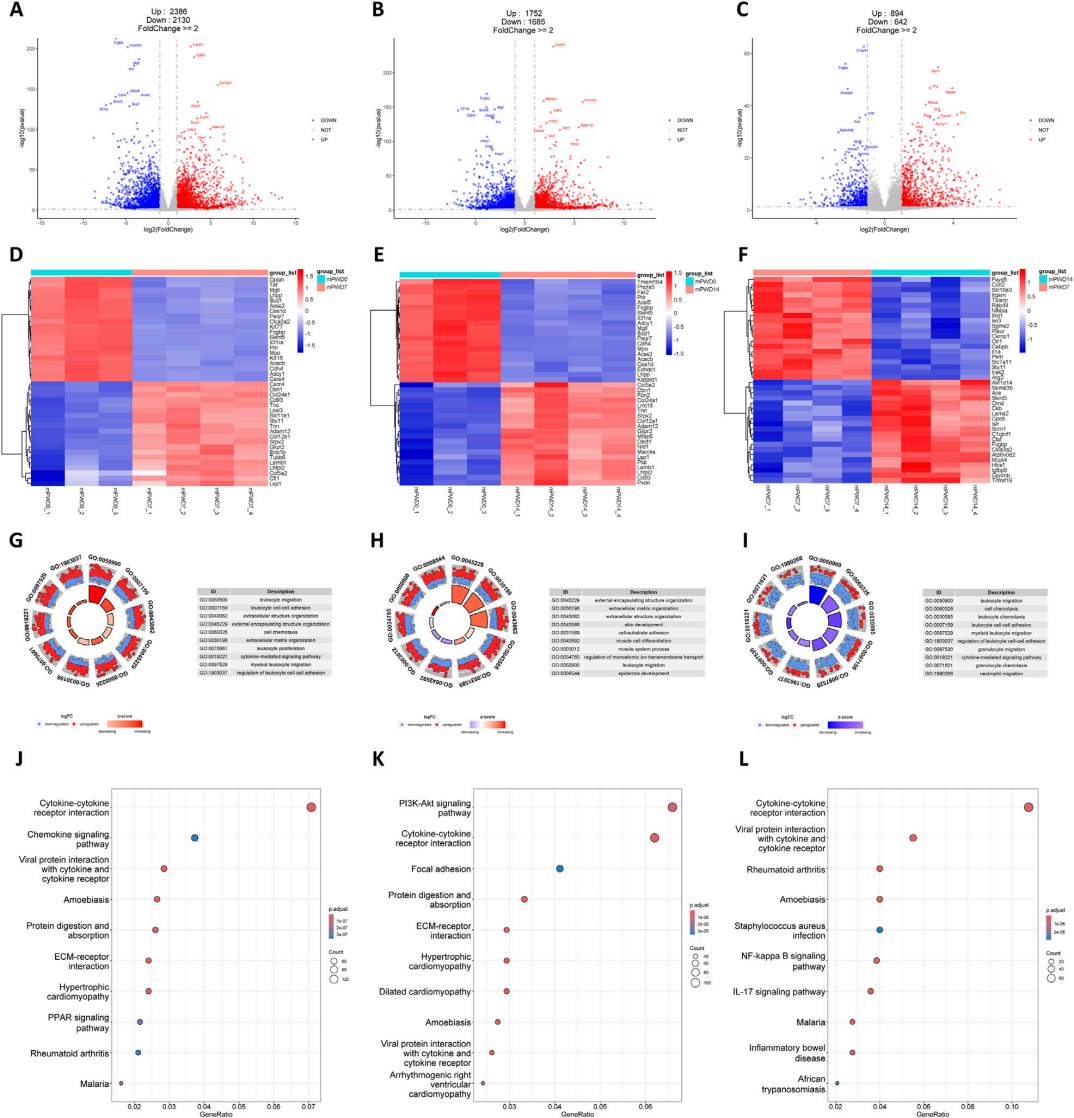
Transcriptomic analysis of mouse during skin wound healing. **(A–C)** Volcano plot of mPWD7 vs mPWD0 **(A)**, mPWD14 vs mPWD0 **(B)**, and mPWD14 vs mPWD7 **(C)** comparison. Red indicates upregulated genes and blue indicates downregulated genes. Top 20 differentially expressed genes are labeled in the map. **(D–E)** Heatmap of top 20 differentially expressed genes in mPWD7 vs mPWD0 **(D)**, mPWD14 vs mPWD0 **(E)**, and mPWD14 vs mPWD7 **(F)** comparison. **(G–I)** Biological process enrichment of differentially expressed genes in mPWD7 vs mPWD0 **(G)**, mPWD14 vs mPWD0 **(H)**, and mPWD14 vs mPWD7 **(I)** comparison. **(J–L)** Kyoto Encyclopedia of Genes and Genomes (KEGG) analysis of differentially expressed genes in mPWD7 vs mPWD0 **(J)**, mPWD14 vs mPWD0 **(K)**, and mPWD14 vs mPWD7 **(L)** comparison.

Similar to the human transcriptome, thousands of DEGs in mPWD7 and mPWD14 compared to mPWD0 were found in mouse transcriptome (N = 4,516 and 3,437, respectively), which also showed more upregulated genes than downregulated genes (Figures 3A, B). While mPWD14 vs mPWD7 had fewer DEGs (N = 1,536) compared to the first two comparison, they were still more abundant than hPWD21 vs hPWD14 comparison in the human transcriptome (Figure 3C). The heatmap revealed numerous DEGs shared between mPWD7 and mPWD14 compared to mPWD0, like Dbn1, Col24a1, Cd93, Tnn, Adam12, Col12a1, Srpx2, Glipr2, Lamb1, Lhfpl2, Col5a2, and Lsp1 with increased expression, and Mgll, Lhpp, Acss2, Ces1d, Paqr7, Fcgbp, Skint5, Il31ra, Prlr, Mpo, Acacb, Cdh4, and Adcy1 with decreased expression (Figures 3D, E). Among them, Skint5 and Fcgbp restored expression in mPWD14, with high expression compared with mPWD7, but low expression compared with mPWD0 (Figure 3F). F). DEGs in mPWD7 and mPWD14 were also enriched in extracellular matrix and leukocyte related process, including external encapsulating structure organization, extracellular matrix organization, extracellular structure organization, and leukocyte migration (Figures 3G, H). The enriched biological processes in the mouse transcriptome mirrored those in humans, suggesting shared molecular mechanisms in skin wound healing across species. While in comparison of mPWD14 and mPWD7, DEGs were only associated with leukocyte related process, no extracellular matrix related process was found (Figure 3I). DEGs enriched in KEGG showed cytokine and cellular homeostasis were main pathways in mPWD7 and mPWD14, like cytokine-cytokine receptor interaction, viral protein interaction with cytokine and cytokine receptor, protein digestion and absorption, and ECM-receptor interaction (Figures 3J, K). And most of DEGs in mPWD14 compared with mPWD7 were associated with cytokine-cytokine receptor interaction (Figure 3L).

### Shared genes of human and mouse in skin wound healing

**FIGURE 4:**
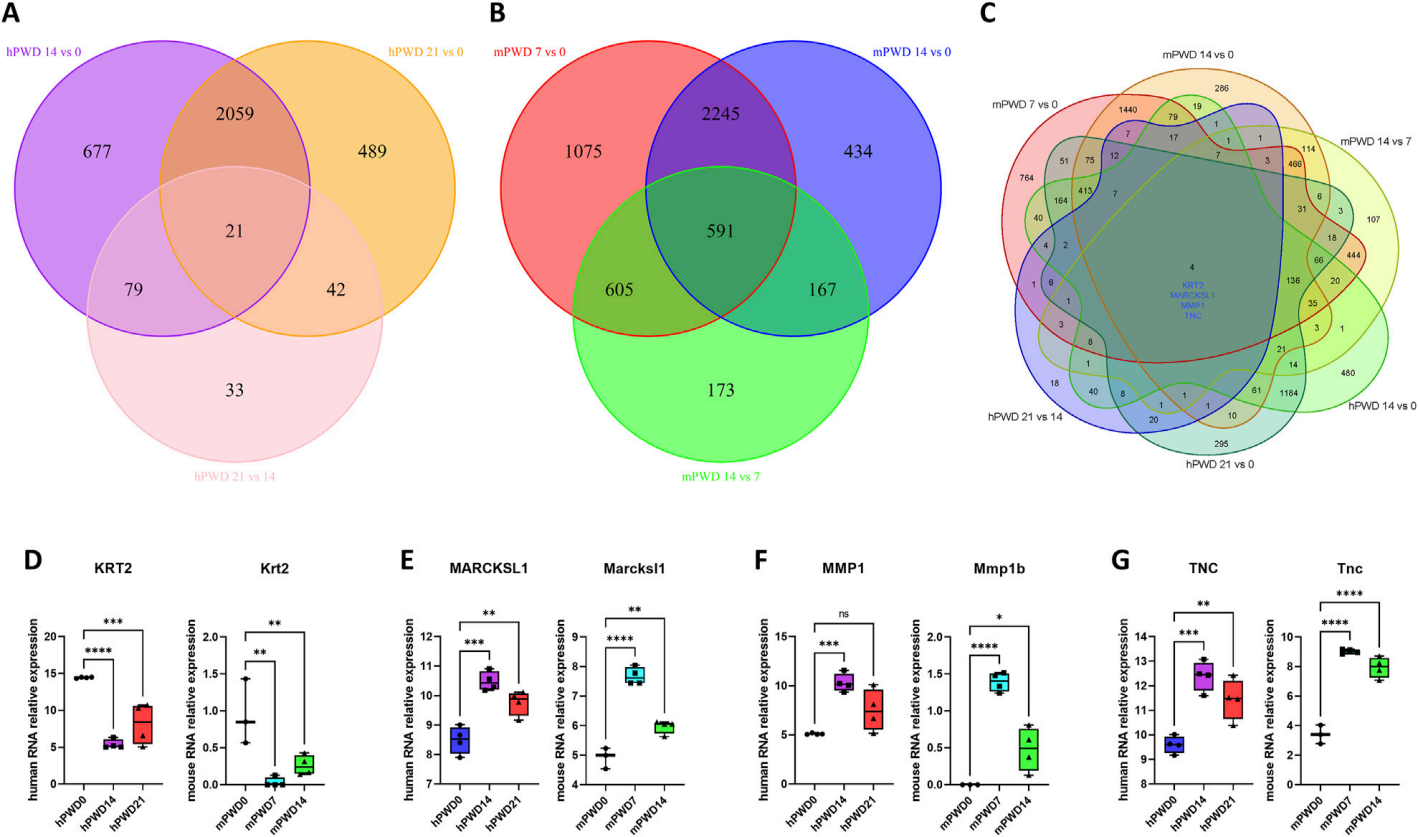
Shared genes of human and mouse in skin wound healing. **(A)** Venn diagram of hPWD14 vs 0, hPWD21 vs 0, and hPWD21 vs 14 in human transcriptomes. **(B)** Venn diagram of mPWD7 vs 0, mPWD14 vs 0, and mPWD14 vs 7 in mouse transcriptomes. **(C)** Venn diagram of six comparisons in human and mouse transcriptomes. Four shared genes show significant differential expression, including KRT2, MARCKSL1, MMP1, and TNC. **(D–G)** The relative expression of four homologous genes KRT2/Krt2 **(D)**, MARCKSL1/Marcksl1 **(E)**, MMP1/Mmp1b **(F)**, and TNC/Tnc **(G)** in three stages of wound healing.

There were 21 common genes with differential expression in the comparison of hPWD14 vs 0, hPWD21 vs 0, and hPWD21 vs 14 in human transcriptome (Figure 4A). While there were 591 common genes with differential expression in the comparison of mPWD7 vs 0, mPWD14 vs 0, and mPWD14 vs 7 in mouse transcriptome (Figure 4B). We converted the mouse gene symbols into homologous genes of human, and then analyzed six groups of comparisons. We found that four differentially expressed genes during the skin wound healing process in both humans and mice, including KRT2, MARCKSL1, MMP1, and TNC (Figure 4C). KRT2 showed a decreasing expression in hPWD14 vs 0 and mPWD7 vs 0 after wound injury, and an increasing expression in hPWD21 vs 14 and mPWD14 vs 7 (Figure 4D). While MARCKSL1, MMP1, and TNC showed the opposite expression pattern with KRT2, which were upregulated in hPWD14 vs 0 and mPWD7 vs 0, and downregulated in hPWD21 vs 14 and mPWD14 vs 7 (Figures 4E–G). Surprisingly, their expression trends were consistent in both humans and mice, which indicated they might play crucial roles in mammalian skin wound healing.

### Molecular network of skin wound healing in human and mouse

**FIGURE 5:**
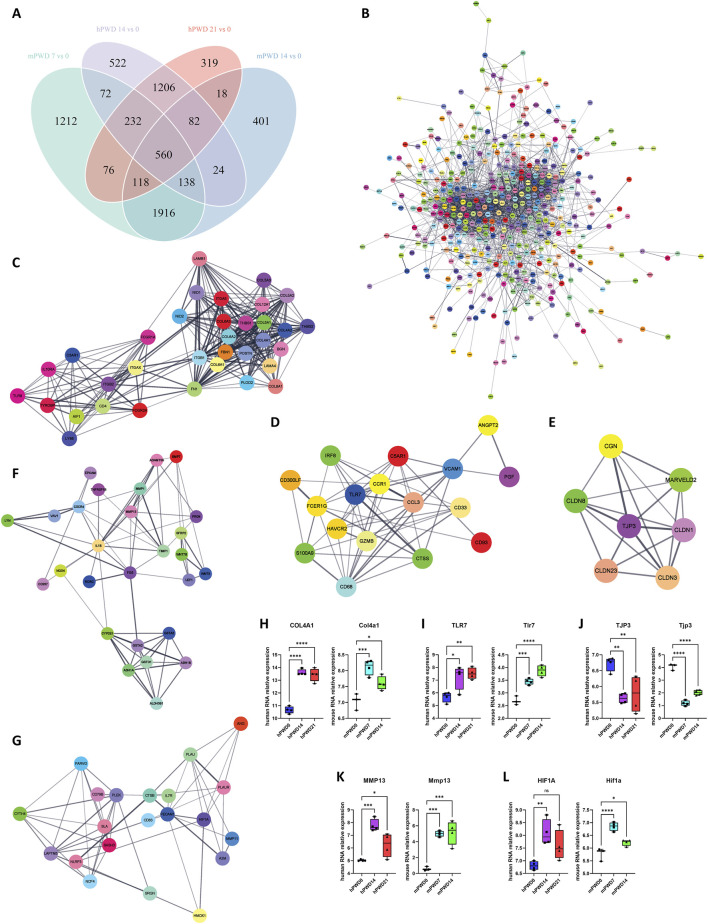
Molecular network of skin wound healing in human and mouse. **(A)** Venn diagram of human and mouse transcriptomes before and after wound healing. **(B)** A total of 560 shared genes are constructed into a protein-protein interaction network. **(C–E)** The main network is further divided into 5 subnetworks, including subnetwork 1 **(C)**, subnetwork 2 **(D)**, subnetwork 3 **(E)**, subnetwork 4 **(F)** and Subnetwork 5 **(G)**. **(H–L)** The relative expression of hub genes of five subnetworks COL4A1/Col4a1 **(H)**, TLR7/Tlr7 **(I)**, TJP3/Tjp3 **(J)**, MMP13/Mmp13 **(K)**, and HIF1A/Hif1a **(L)** in three stages of skin wound healing.

In order to understand the molecular mechanism of skin wound healing, we investigated the differentially expressed genes pre- and post-injury in humans and mice in human and mice. A total of 560 genes underwent expression changes before and after injury in human and mice (Figure 5A). These genes were imported in the STRING database to construct a protein-protein interaction network (Figure 5B). Using Cytoscape software MCODE plugin, we further decomposed this network into 5 subnetworks (Figures 5C–G). Subnetwork 1 was mainly associated with collagen synthesis, including types IV, V, VI, VIII, and XII collagen (Figure 5C). Subnetwork 2 was mainly related to immunity, including TLR7, CTSS, and CCR1 (Figure 5D). Subnetwork 3 was associated with cell-cell adhesion, including TJP3 and Claudin family members (Figure 5E). Subnetwork 4 was mainly related with extracellular matrix breakdown caused by inflammation, including inflammatory factors IL1B, NOX4, and matrix metalloproteinases MMP1, MMP13, ADAMTS5 (Figure 5F). Subnetwork 5 was also mainly associated with immunity, including HIF1A, HMOX1, and NLRP3 (Figure 5G). Some hub genes such as COL4A1, TLR7, TJP3, MMP13, and HIF1A exhibited significant expression changes before and after wound injury in human and mice (Figures 5H–L). COL4A1, TLR7, MMP13, and HIF1A showed an increased expression, while TJP3 showed a decreased expression after wound injury. These results indicated that metabolism of extracellular matrix and immune response were enhanced after wound injury, while cell-cell adhesion was weakened.

## Discussion

Skin wound healing is a complex and dynamic process that involves various cellular components and tissues, with a series of signaling pathways contributing distinct functions at each phase of the healing process, which are essential for the restoration of tissue integrity following injury. We used transcriptomic data from human and mouse models across three distinct stages of skin wound healing to identify shared differentially expressed genes (DEGs) and constructed detailed molecular networks. This approach provided valuable insights into the common molecular mechanisms underlying skin wound healing in both species.

By normalizing the transcriptomes and performing PCA and clustering, we observed distinct transcriptional profiles that correspond to different stages of skin wound healing. The human transcriptomes showed differences before and after wound injury and a degree of similarity between the later stages of skin wound healing (hPWD14 and hPWD21), indicating differences in molecular processes involved before and after wound injury and convergence of molecular processes involved in two and 3 weeks of wound injury. In contrast, the mouse transcriptomes (mPWD0, mPWD7, mPWD14) exhibited more significant differences, indicative of a more heterogeneous response to skin wound healing.

The identification of a substantial number of DEGs in the human transcriptome underscored the dynamic changes of the healing process, with a notable upregulation of genes involved in extracellular matrix organization and leukocyte migration. Particularly those gene expression changes before and after wound injury, pointed to the activation of critical pathways involved in tissue repair and inflammation management. The increased expression of genes such as COL6A3, THY1, and MMP11, which were known to play roles in matrix remodeling and immune cell recruitment, was consistent with the active phases of wound healing characterized by tissue formation and repair (Zhu et al., 2021; Pérez et al., 2022; Caley et al., 2015). Interestingly, the downregulation of genes in the hPWD21 vs hPWD14 comparison suggested a possible attenuation of the healing response after 3 weeks of wound injury, which might be associated with infection as indicated by the KEGG analysis.

In the mouse transcriptome, a similar pattern of DEGs was observed. The predominance of upregulated genes over downregulated genes in the mPWD7 and mPWD14 comparisons to mPWD0 suggested an active phase of cellular response and tissue regeneration. The heatmap analysis highlighting shared DEGs between mPWD7 and mPWD14 compared to mPWD0 pointed to the involvement of specific genes in the skin wound healing process. Notably, the restored expression observed for genes such as Skint5 and Fcgbp in mPWD14 indicated a potential role in the resolution of inflammation and the initiation of tissue remodeling (Keyes et al., 2016; Gorman et al., 2023). The enrichment of DEGs in processes related to the extracellular matrix and leukocyte migration further emphasized the importance of these biological pathways in mediating the skin wound healing response. The observation that DEGs in the comparison of mPWD14 and mPWD7 were primarily associated with leukocyte-related processes, rather than extracellular matrix-related processes, suggested a shift in the healing process from tissue formation to immune modulation. This was supported by the KEGG enrichment analysis, which identified cytokine and cellular homeostasis as the main pathways in mPWD7 and mPWD14, reflecting the dynamic interplay between immune response and tissue repair.

The identification of shared DEGs between human and mouse transcriptomes, such as KRT2, MARCKSL1, MMP1, and TNC, provided compelling evidence for the conservation of skin wound healing mechanisms across species (Fischer et al., 2016; Liang et al., 2020; Keskin et al., 2021; Wang et al., 2022). The consistent expression trends of these genes in both humans and mice suggested that they might play essential roles in the skin wound healing process. The upregulation and subsequent downregulation of MARCKSL1, MMP1, and TNC, and the inverse expression pattern of KRT2, indicated the temporal regulation of these genes and their potential involvement in different stages of skin wound healing.

The construction of a protein-protein interaction network from the differentially expressed genes in both species allowed us to identify five distinct subnetworks that were pivotal to the skin wound healing process. These subnetworks were involved in collagen synthesis, immune response, cell-cell adhesion, and extracellular matrix, which were critical components of the healing cascade. The significant expression changes in hub genes such as COL4A1, TLR7, TJP3, MMP13 and HIF1A after wound injury further highlighted the importance of these molecules in modulating the healing process (Huebener and Schwabe, 2013; Hattori et al., 2009; Deschene et al., 2012; Du et al., 2021).

There were several limitations that must be acknowledged. Firstly, the different skin wound healing mechanism in humans and mice might partially affect the comparative analysis results, as humans heal from granulation tissue formation and mice heal from subcutaneous muscle contraction (Wong et al., 2011). Secondly, the different stages of hair follicles in mice might influence the comparison, but this could be distinguished by differences in the expression of hair follicle related genes. For example, Foxn1 plays a crucial role in hair follicle development (Mecklenburg et al., 2001), and its expression was upregulated in mPWD7 and 14 mouse wound models, while no differences were found in human wound models. Thirdly, mouse transcriptome was obtained from RNA-seq, while the human transcriptome was used chip detection technology, which might lead to the loss of certain low abundance expressed genes in humans. Fourthly, the results were relied on existing transcriptome datasets which limited the analysis of the specific time points and conditions. The transcriptomes were mainly at tissue remodeling stage (Zlobina et al., 2023), which might not fully represent the entire wound healing process, as it was a highly complex and dynamic event that might involve additional temporal and spatial factors. Fifthly, the individual variability in human samples and the potential for minimal transcriptomic changes in the later stages of healing could introduce variability in the results, potentially affecting the accuracy of the identified DEGs. Lastly, we identified shared and unique molecular mechanisms, but the direct clinical applicability of these findings required further validation through functional studies. Despite these limitations, we laid a solid foundation for future research aimed at improving skin wound healing outcomes through a better understanding of the underlying molecular processes.

In conclusion, our transcriptomic analysis provided insights to the shared and distinct molecular mechanisms underlying skin wound healing in humans and mice. The identification of conserved genes and networks not only provided a deeper understanding of the healing process but also offered potential targets for therapeutic intervention.

## Data Availability

The original contributions presented in the study are included in the article/Supplementary Material, further inquiries can be directed to the corresponding authors.
