# Supplementary figures and images for: Comparative analysis of human and mouse transcriptomes during skin wound healing
Source: Front Cell Dev Biol. 2024 Oct 29;12:1486493. doi: 10.3389/fcell.2024.1486493 (PMC11554618; doi:10.3389/fcell.2024.1486493)

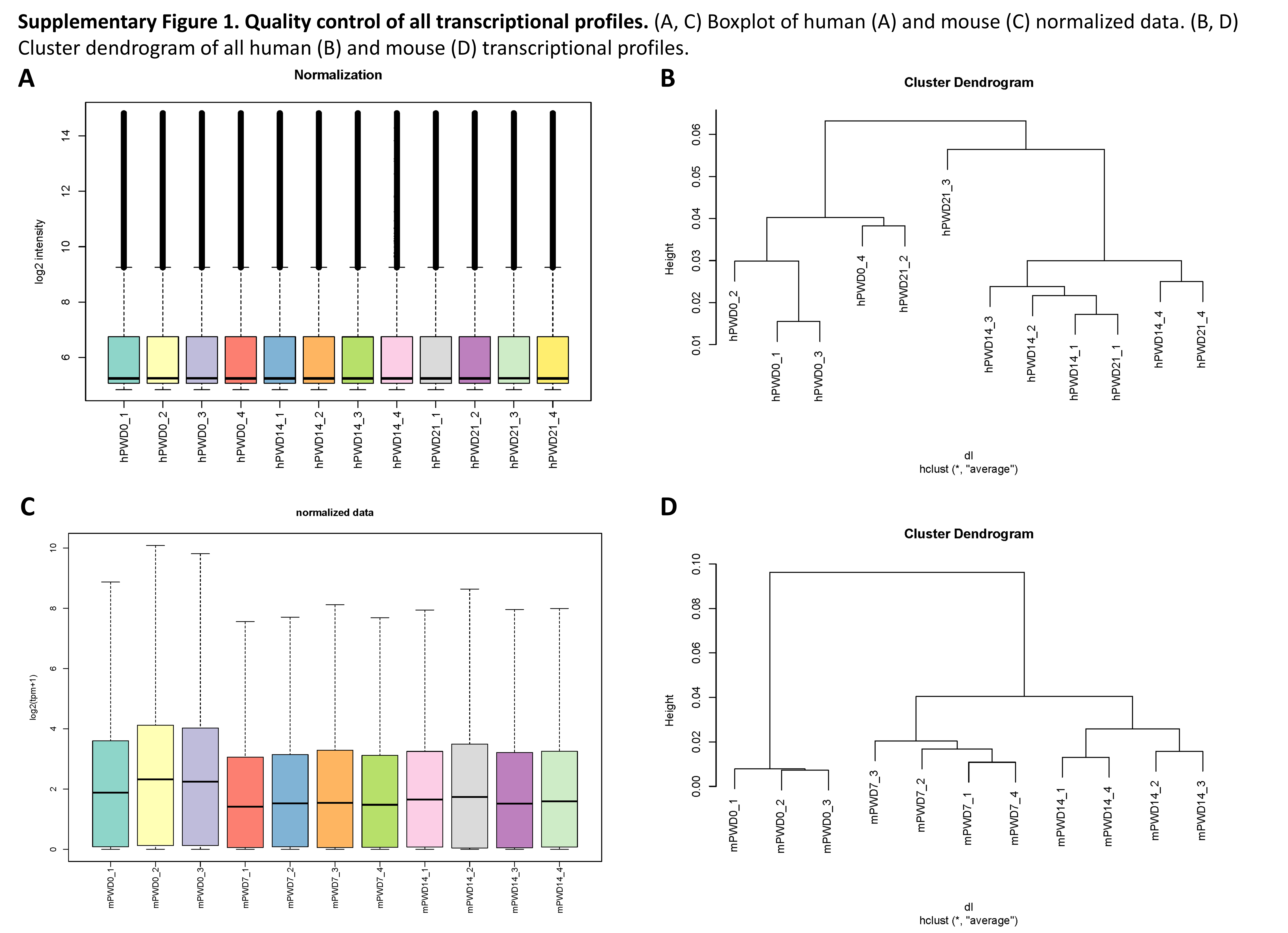

Supplement: Supplementary file 1 [file Image1.tif]
